# Selective binding of virulence type III export chaperones by FliJ escort orthologues InvI and YscO

**DOI:** 10.1111/j.1574-6968.2009.01535.x

**Published:** 2009-03-02

**Authors:** Lewis DB Evans, Colin Hughes

**Affiliations:** Department of Pathology, Cambridge UniversityCambridge, UK

**Keywords:** type III export, secretion pilots, chaperone escort

## Abstract

Bacteria secrete flagella subunits and deliver virulence effectors via type III export systems. During flagellar filament assembly, a chaperone escort mechanism has been proposed to enhance the export of early, minor flagellar filament components by selectively binding and cycling their chaperones. Here we identify virulence orthologues of the flagellar chaperone escort FliJ and show that the orthologues *Salmonella* InvI and *Yersinia* YscO are, like FliJ, essential for their type III export pathway and similarly, do not bind export substrates. Like FliJ, they recognize a subset of export chaperones, in particular those of the host membrane translocon components required for subsequent effector delivery.

## Introduction

Type III secretion systems enable Gram-negative bacteria to assemble cell surface flagella (Aizawa, 2001) and deliver virulence effectors to eukaryotic cells (Galan & Wolf-Watz, 2006). The integral membrane components of the export machineries are closely related (Kubori *et al.*, 1998), and in both cases cytosolic chaperones pilot cognate late export substrates to dock at the membrane-associated ATPase complex (Auvray *et al.*, 2002; Gauthier & Finlay, 2003; Thomas *et al.*, 2004; Akeda & Galan, 2005). The export processes are ordered: the flagellar basal body/rod/hook substructures are assembled before filament subunits are exported, while completion of the virulence needle complex initiates export and assembly of the translocon into the eukaryotic cell membrane to allow delivery of effectors (Homma *et al.*, 1984; Sukhan *et al.*, 2001). This order is governed in part by a substrate specificity switch from rod/hook to filament subunits or from needle to translocon components and effectors (Fraser *et al.*, 2003; Sorg *et al.*, 2007), during which early components and late export substrates are suggested to be sorted after docking at each export machinery (Stafford *et al.*, 2007; Riordan & Schneewind, 2008). We have proposed that an additional mechanism could operate during the late export of flagellar filament subunits, as the minor filament substructures (the hook–filament junction and filament cap) must assemble before the large number of major filament (flagellin) subunits can be incorporated (Homma *et al.*, 1984). We have reported that a novel escort protein FliJ located in the export ATPase complex at the base of the export apparatus can recruit and cycle-free unloaded chaperones of minor substructure subunits. As FliJ does not recognize the chaperone of the major subunit flagellin (Evans *et al.*, 2006), we proposed that it could preferentially enhance the formation of minor chaperone–subunit complexes and thus favour assembly of the filament junction and cap. This would be beneficial as these minor subunits are thought to compete for export with the major subunit (Homma *et al.*, 1984; Kubori *et al.*, 1998).

Here, we assess whether a similar FliJ-like chaperone escort activity could operate late in the virulence type III pathway, i.e. after needle complex assembly. We assessed whether the putative FliJ orthologues *Salmonella typhimurium* InvI and *Yersinia enterocolitica* YscO could similarly selectively recognize free chaperones that facilitate export of late substrates destined for the host cell.

## Materials and methods

### Bacterial strains and plasmids

Bacteria were cultured at 37 °C to the late exponential phase (A_600 nm_ 2.0), unless stated, in Luria–Bertani broth containing, where appropriate, ampicillin, chloramphenicol or kanamycin (at 50–20 μg mL^−1^). *In vivo* studies were performed in wild-type *S. typhimurium* SJW1103, and chromosomal deletion mutant *invI*::*K*_m_^R^, in which the gene was replaced by a kanamycin resistance cassette, was constructed using the Red recombinase system (Datsenko & Wanner, 2000). Recombinant proteins were expressed in *Escherichia coli* C41 from isopropyl-β-d-thiogalactoside-inducible plasmids.

Recombinant plasmids encoding individual virulence chaperones, export substrates and putative FliJ orthologues genes were constructed by PCR amplification using *Pfu* turbo DNA polymerase from *S. typhimurium* genomic DNA and *Y. enterocolitica* virulence plasmid pYVe227. To make glutathione-*S*-transferase (GST) fusions of InvI and YscO, genes were amplified by PCR, and products were inserted BamHI/XhoI into pGEX-4T-3 (GE Healthcare). PCR products of *Salmonella sicA, invB, sigE* and *sicP* and *Yersinia sycD, sycE, sycH, sycO, sycT, yopD, yopE, yopH, yopO* and *yopT* were inserted either NdeI/BamHI or NdeI/HindIII into pACT7 (Kaelin *et al.*, 1992) or pET15b (Novagen). Histidine-tagged recombinant plasmids of *Salmonella* virulence genes (*sipA, sipB, sipC, sipD, sopE* and *sptP*) were gifted by the Koronakis laboratory. Inserts were verified by DNA sequencing. Recombinant genes encoding InvI (and N/C terminally histidine-tagged InvI) were constructed by PCR and inserted XbaI/HindIII into pBAD18 (Guzman *et al.*, 1995).

### Purification of proteins

Detailed purification protocols have been published previously (Hayward & Koronakis, 1999; Hayward *et al.*, 2000; McGhie *et al.*, 2001). In brief, cells expressing individual histidine-tagged recombinant proteins were resuspended in buffer A containing phosphate buffer, NaCl and detergent [50 mM NaH_2_PO_4_ (pH 7.4–8.6), 150–300 mM NaCl, 1 mM dithiothreitol and 0–0.5% Triton X-100 (v/v)], before lysis in a French pressure cell (Aminco). Cleared cell lysates were passed over nickel nitrilotriacetic acid (N^2+^) agarose (Qiagen) and recombinant proteins were eluted using imidazole. Recombinant proteins SipB, SipC, YopD, YopT and YopE were purified under denaturing conditions (6 M guanidinium chloride) from the insoluble fractions and elution fractions were dialysed in series first, against buffer A containing 0.5 M pyridinio propanesulphonate, followed by buffer A alone.

### Affinity chromatography copurification assays

Copurification of protein complexes was achieved with either N^2+^ agarose or glutathione sepharose 4B as described previously (Evans *et al.*, 2006). Chaperone prey proteins were native, whereas purified effector prey proteins (SipA, SipB, SipC, SipD, SopE, SptP, YopD, YopE, YopH, YopO and YopT) were histidine tagged. *In vitro* mixed purified proteins or cleared cell lysates were incubated for 2 h with affinity resin. After extensive washing [buffer A (10–60 mM imidazole)], proteins were eluted in sodium dodecyl sulfate (SDS) loading buffer. For *in vivo* studies, soluble lysates of *S. typhimurium* strains expressing His-InvI at an export complementing level from arabinose (0.01%)-inducible plasmid pBAD18 (Guzman *et al.*, 1995) were prepared as above, incubated for 1 h with N^2+^ agarose, washed three times with buffer A (60 mM imidazole) and proteins eluted in SDS loading buffer; untagged InvI was used as a negative control.

### Assay of virulence effector protein export

*Salmonella typhimurium* culture supernatants were clarified by centrifugation and passed through a 0.2-μm nitrocellulose filter (Millipore). Virulence proteins were precipitated by 10% (v/v) trichloroacetic acid on ice for 1 h, separated by SDS-polyacrylamide gel electrophoresis (SDS-PAGE) and visualized by immunoblotting with appropriate polyclonal antisera (Cain *et al.*, 2004).

## Results and discussion

**Figure 1:**
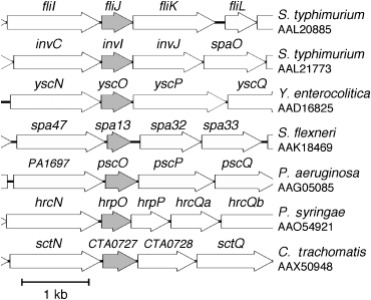
Selected virulence type III secretion operons aligned using COLIBASE (http://xbase.bham.ac.uk/), TIGR (http://www.tigr.org/) and NCBI (http://www.ncbi.nlm.nih.gov/) to the *Salmonella typhimurium* LT2 *fliF*-*K* flagellar operon. These contain the chaperone escort gene *fliJ* located between the ATPase (*fliI*) and hook length control (*fliK)* genes.

While the bacterial type III export membrane components are generally obvious homologues, this is not so for the chaperones (Bennett & Hughes, 2000), which in the virulence systems bind effector N-terminal regions (Page & Parsot, 2002) rather than the flagella subunits C-terminal polymerization domains (Auvray *et al.*, 2001). Nevertheless, virulence operons contain an essential (Collazo *et al.*, 1995; Payne & Straley, 1998) but currently anonymous gene that, like *fliJ*, lies between the genes encoding the export ATPase and a protein known or suspected to control hook or needle length (Journet *et al.*, 2003; Shibata *et al.*, 2007) (Fig. 1). These virulence genes encode, in each case, a protein of a size similar to FliJ (14–18 kDa), and while these do not show significant sequence similarity to FliJ, they are predicted to have comparably high helicity (data not shown).

**Figure 2:**
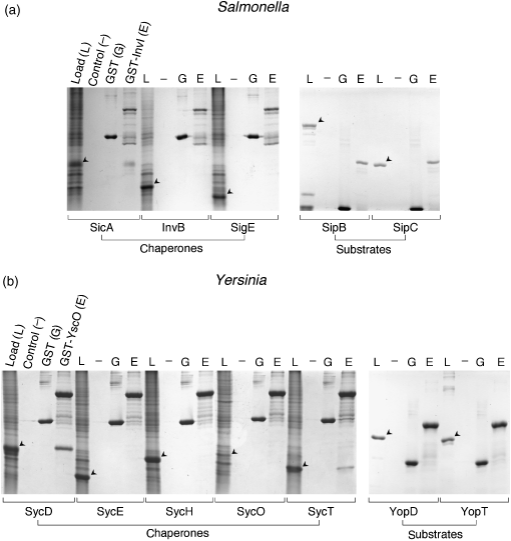
Affinity chromatography following incubation with (E) and without (−) GST-tagged FliJ orthologue (InvI or YscO) or with unfused GST (G). (a) *Salmonella* InvI or (b) *Yersinia* YscO with cell lysates (load L) from *Escherichia coli* C41 expressing the respective *Salmonella* and *Yersinia* chaperones and substrates (indicated by arrows). Samples were separated by SDS-PAGE (10/15%) and stained with Coomassie blue.

To establish whether *S. typhimurium* InvI and *Y. enterocolitica* YscO proteins could have a chaperone escort function analogous to FliJ, we applied *in vitro* affinity chromatography to identify possible recognition of free unloaded chaperones that facilitate export of late substrates destined for the host cell. A representative set of late virulence chaperones was incubated with (E) and without (−) GST-InvI (46 kDa) or *Yersinia* YscO (46.6 kDa) with glutathione sepharose. Figure 2a shows that *Salmonella* GST-InvI bound the chaperone SicA (19 kDa), but not chaperones InvB (15 kDa) or SigE (13 kDa); SicP was poorly expressed and excluded from the study. Similarly (Fig. 2b), *Yersinia* GST-YscO recognized chaperone SycD (19 kDa), and less prominently the chaperone SycT (15 kDa), but not chaperones SycE (15 kDa), SycH (16 kDa) or SycO (17 kDa). We also assessed the ability of these putative escort orthologues to bind cognate partners of the recognized chaperones, as previously FliJ was reported to have general chaperone activity thought to interact with subunits of the flagellum (Minamino *et al.*, 2000). These interactions were not detected in assays where escort–chaperone interactions were elucidated (Evans *et al.*, 2006). Figure 2 shows that neither of the purified cognate partners of SicA, SipB (62 kDa) or SipC (42 kDa) bound to GST-InvI. *Yersinia* GST-YscO (Fig. 2) was also unable to recognize the purified cognate-binding partners of either SycD (YopD, 33 kDa; YopB was poorly expressed and excluded from the study) or SycT (YopT, 36 kDa). None of the other effectors tested, SipA, SipD, SopE, SptP, YopE, YopH or YopO, bound their respective FliJ orthologues (Supporting Information, Fig. S1). This indicates that InvI and YscO are not general chaperones. Purified cognate substrates assayed still bound their chaperones (Fig. S1), and no chaperone, translocon component or effector bound unfused GST (G) or glutathione sepharose alone (−) (Fig. 2).

**Figure 3:**
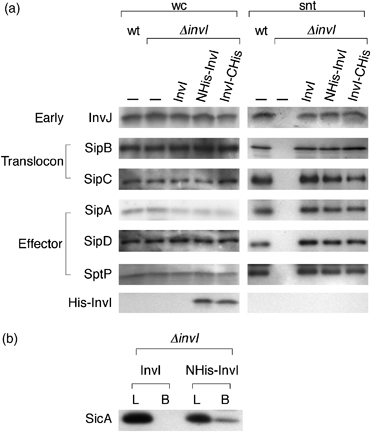
*In vivo* activity of InvI. (a) Export of virulence substrates by *Salmonella* wild type (wt) and *Salmonella invI* deletion mutant (Δ*invI*) with InvI variants (InvI, NHis-InvI and InvI-CHis) *in trans* and without (−). Secretion was assayed by SDS-PAGE and immunoblotting for early needle length control protein (InvJ) and translocon components (SipB and SipC) and effectors (SipA, SipD and SptP) in concentrated supernatants (snt) from late exponential Luria–Bertani cultures (wc, whole culture). Export of N and C terminally histidine-tagged variants *in trans* (nHis-InvI and InvI-cHis) was also assayed in the *Salmonella invI* deletion mutant using a Tetra-His antibody (Qiagen). (b) *In vivo* isolation (bound, B) of wild-type SicA chaperone by functional histidine-tagged InvI. Affinity chromatography of lysates (L) from *Salmonella invI* deletion strain (Δ*invI*) expressing either control InvI or histidine-tagged InvI as above. Samples were separated by SDS-PAGE (15%) and immunoblotted.

We assessed export by *S. typhimurium* SJW1103 after replacing the chromosomal *invI* gene with a kanamycin resistance cassette (Datsenko & Wanner, 2000). Figure 3a shows the whole cell (wc) and supernatant (snt) of cultures of the wild type (wt) and Δ*invI* mutant. The mutation severely disabled export of all export substrates tested, including early needle length control protein InvJ (Journet *et al.*, 2003), as well as the translocon components SipB and SipC, and the effectors SipA, SipD and SptP. The export of all these substrates was recovered by the addition of InvI, *in trans*, induced with 0.01% arabinose. This agrees with data showing that an *invI* mutation attenuates *Salmonella* entry into host cells (Collazo *et al.*, 1995), and *yscO* mutations disable type III effector export (Payne & Straley, 1998). These data are also compatible with the *fliJ* mutant (Minamino *et al.*, 2000) that attenuates export of unchaperoned early and chaperoned late subunits. Attenuated secretion of early component InvJ protein does not negate the possibility of needle assembly in a Δ*invI* mutant. However, these findings suggest that FliJ orthologues may have an additional role before the late chaperone escort activity. FliJ increases ATP hydrolysis of the membrane-associated export ATPase FliI (Evans *et al.*, 2006), and YscO has been copurified with blocked export machinery complexes containing the ATPase YscN (Riordan & Schneewind, 2008). Like FliJ, no N or C terminally histidine-tagged InvI was detected (by immunoblotting, Fig. 3a) in the supernatant fraction, in contrast with an observation that YscO might be exported (Payne & Straley, 1998). These data provide evidence that escort orthologue proteins form part of the membrane export machinery and may help explain the global effect on secretion. Finally, the cell lysate immunoblot of Fig. 3b shows that NHis-InvI (which similarly complemented the *invI* mutant defect, Fig. 3a) copurified with the SicA chaperone, confirming the formation of an *in vivo* complex with its specific chaperone target.

The data show that, like the flagellar escort FliJ, InvI is essential for chaperoned and unchaperoned export, and InvI and YscO do not bind export substrates and recognize a subset of export chaperones. We could not demonstrate the competitive acquisition of escort-bound chaperones by cognate substrates evident in the flagellar system (Evans *et al.*, 2006). Also, we cannot rule out the possibility of a tripartite complex of escort–chaperone and effector. The results nevertheless provide support for the possibility that an FliJ-like escort mechanism may similarly allow selective cycling of virulence chaperones. What might be the advantage of this? Although the significance of the weak YscO interaction with SycT is unclear [the SycT partner YopT is a cysteine protease effector (Aepfelbacher *et al.*, 2003)], both InvI and YscO bind the chaperones unequivocally for the respective translocon components. Chaperone SicA binds translocon component SipC (Tucker & Galan, 2000) and possibly SipB (Kaniga *et al.*, 1995), while SycD chaperones the translocon components YopB and YopD (Neyt & Cornelis, 1999a, b). Recent reports propose the notion of ordered export of late substrates (postcompletion of the needle complex) in the virulence system (Sorg *et al.*, 2007). The SipB/C and YopB/D translocons are believed to insert into the host cell membrane and are essential for delivery of effectors destined for the interior of the host cell (Neyt & Cornelis, 1999a, b; Page *et al.*, 1999; McGhie *et al.*, 2002). Preferential export of these membrane translocon components could increase the efficiency of effector delivery. Our data are compatible with an FliJ-like escort function for InvI and YscO, selectively recruiting translocon chaperones to enhance delivery of their cognate-binding partners.

## Supporting Information

Fig. S1. Affinity chromatography of effectorswith (+) and without (-) GST- InvI or GST-YscO as in figure 2.**A.** *Salmonella* SipA (73.9kDa), SipD (37kDa),SopE (26kDa) or SptP (60kDa) or **B.** *Yersinia*YopE (22.9kDa), YopH (50.9kDa) or YopO (81kDa). Affinitychromatography of recombinant chaperones with purified cognatetranslocon components (+hiseffector) or without (control)translocon components. **C.** *Salmonella* SicAchaperone (19kDa) **D.** Yersinia SycD (19kDa) and SycT chaperones (15kDa) (indicated by arrows). Samples were separated by SDS PAGE (10/15%) and stained with Coomassie blue.
