# Supplementary figures and images for: Selective binding of virulence type III export chaperones by FliJ escort orthologues InvI and YscO
Source: FEMS Microbiol Lett. 2009 Mar 2;293(2):292–7. doi: 10.1111/j.1574-6968.2009.01535.x (PMC3500872; doi:10.1111/j.1574-6968.2009.01535.x)

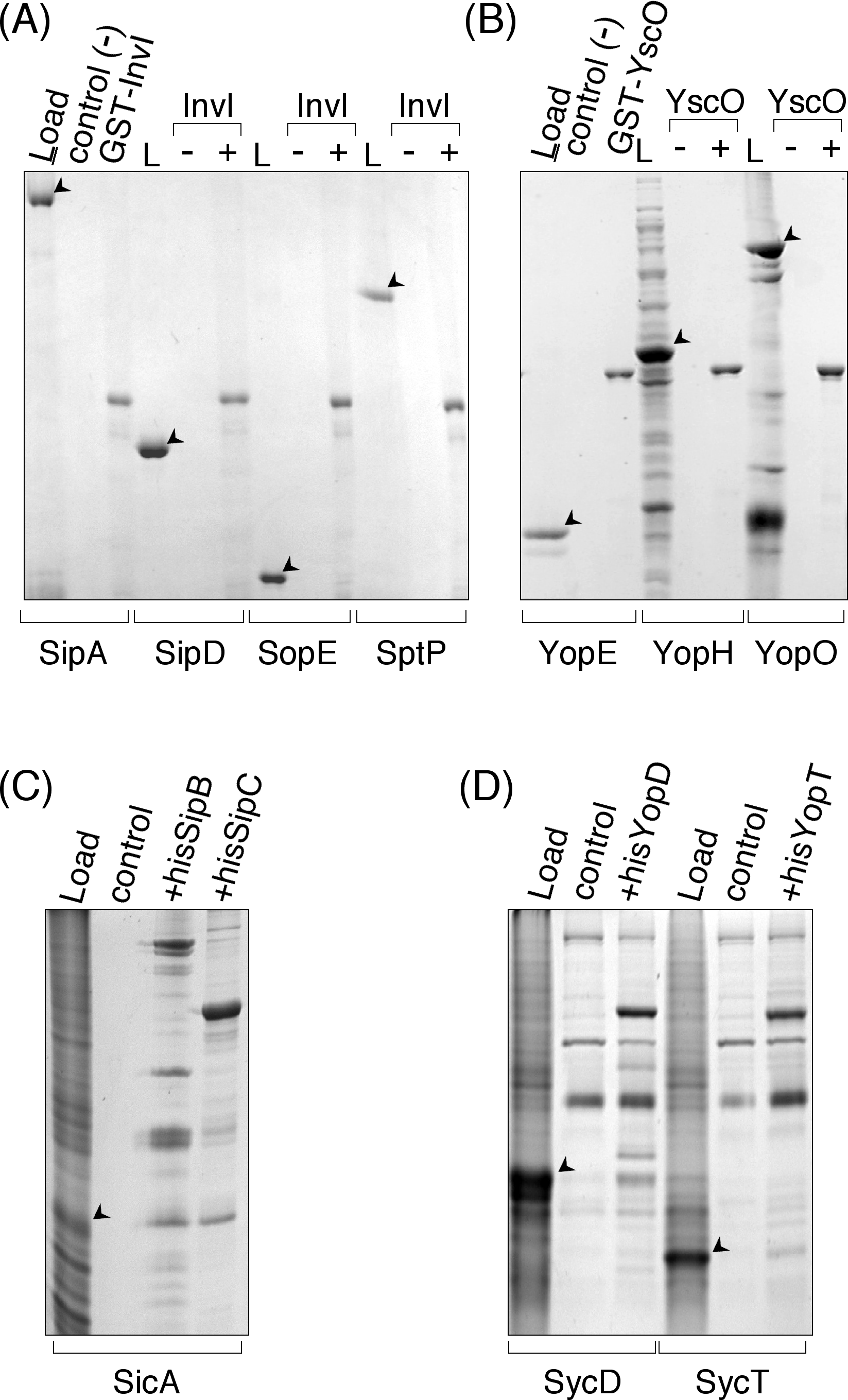

Supplement: Fig. S1. — Affinity chromatography of effectorswith (+) and without (-) GST- InvI or GST-YscO as in figure 2.A. Salmonella SipA (73.9kDa), SipD (37kDa),SopE (26kDa) or SptP (60kDa) or B. YersiniaYopE (22.9kDa), YopH (50.9kDa) or YopO (81kDa). Affinitychromatography of recombinant chaperones with purified cognatetranslocon components (+hiseffector) or without (control)translocon components. C. Salmonella SicAchaperone (19kDa) D. Yersinia SycD (19kDa) and SycT chaperones (15kDa) (indicated by arrows). Samples were separated by SDS PAGE (10/15%) and stained with Coomassie blue. [file fml0293-0292-sd1.tif]
